# Supplementary figures and images for: Increasing neural network robustness improves match to macaque V1 eigenspectrum, spatial frequency preference and predictivity
Source: PLoS Comput Biol. 2022 Jan 7;18(1):e1009739. doi: 10.1371/journal.pcbi.1009739 (PMC8775238; doi:10.1371/journal.pcbi.1009739)

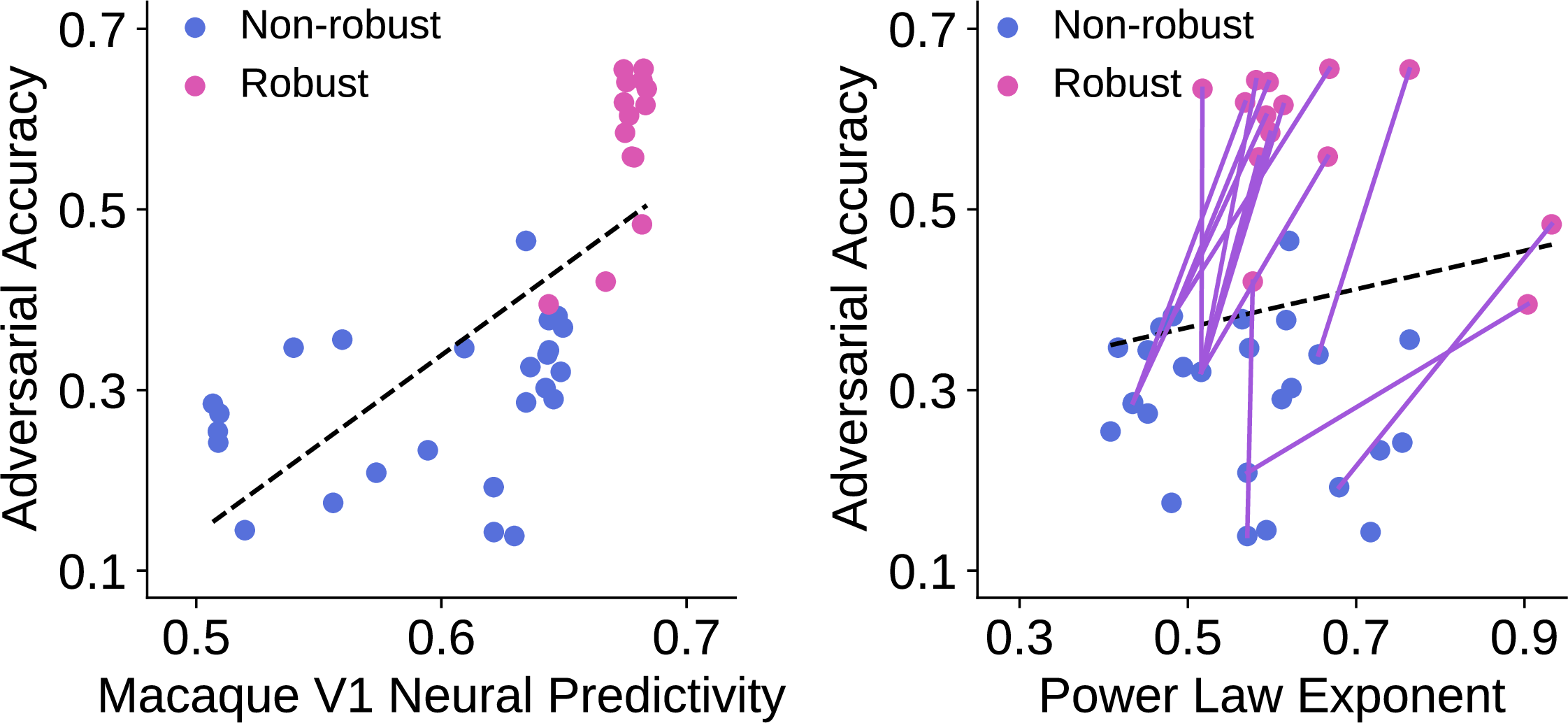

Supplement: S1 Fig — In the main text, we showed results where the “V1-like” layer of a model was obtained via partial least squares regression, where we found that adversarial accuracy correlated with V1 neural predictivity and weakly correlated with power law exponent (recall that the power law exponent for a model was obtained from the model layer that best predicted the macaque V1 neural responses). Here, we show the same figures, but with results obtained via cross-validated ridge regression (where five-fold cross-validation was used to obtain the optimal regularization coefficient). Consistent with our partial least squares regression finding, adversarial accuracy was correlated with V1 neural predictivity (R = 0.696, p < 0.001). However, when using ridge regression to determine the most “V1-like” model layer, adversarial accuracy was found not to correlate with power law exponent (R = 0.159, p = 0.327). Although there was no linear relationship between adversarial accuracy and power law exponent, we noticed that when comparing two models obtained by training a single architecture with and without robustness penalties, the robust model had higher power law exponents (as shown by the purple lines pointing to the upper right in the figure, indicating higher adversarial accuracy and higher power law exponent). This is consistent with the theory of Stringer et al. [26]. (TIFF) [file pcbi.1009739.s001.tiff]

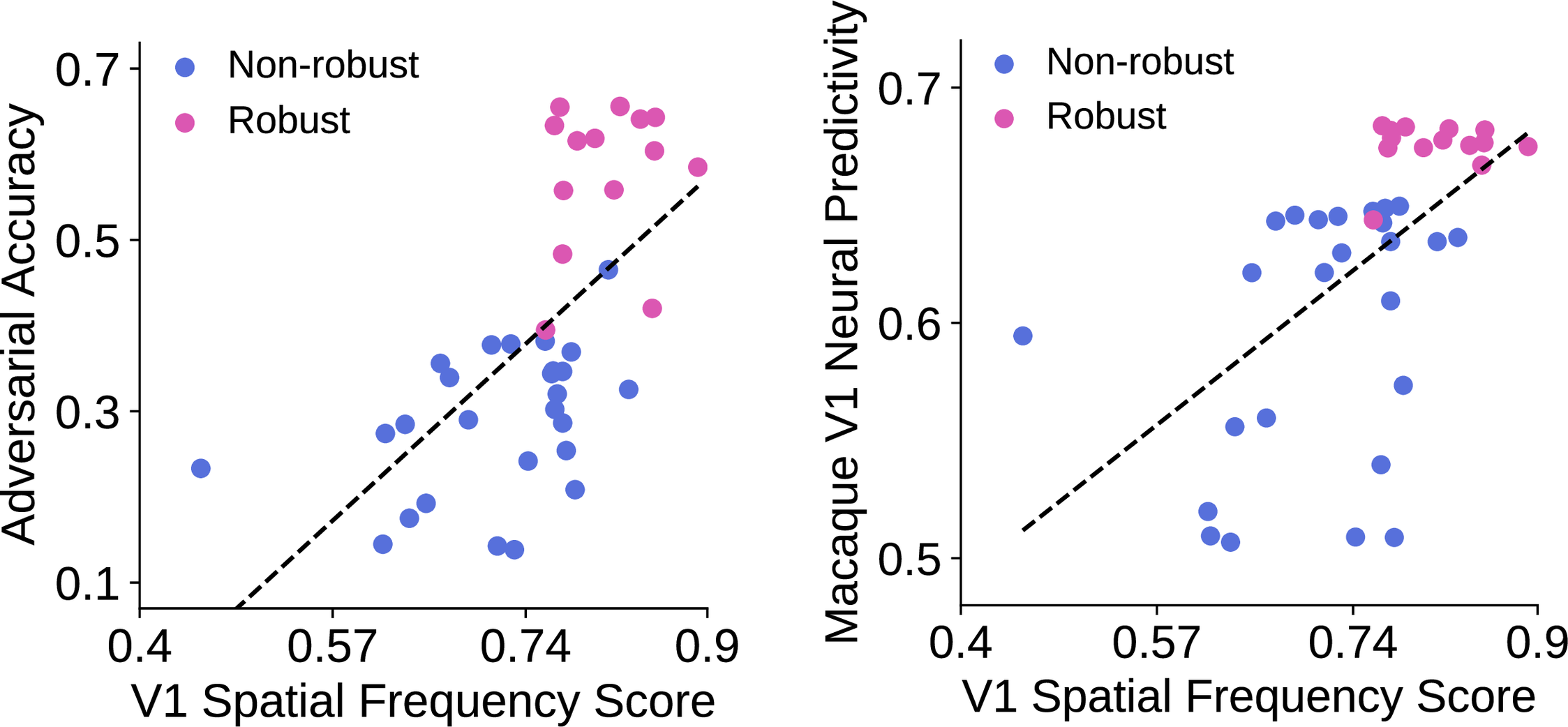

Supplement: S2 Fig — In the main text, we showed the relationship between a model’s adversarial accuracy, its V1 spatial frequency score and its maximum V1 neural predictivity when partial least squares regression was used to obtain a model’s “V1-like” layer. Here, we show results pertaining to these relationships, obtained via cross-validated ridge regression. Qualitatively, the results are the same as those described in the main text. Here, we find that a model’s adversarial accuracy and its V1 spatial frequency score was correlated (R = 0.624, p < 0.001). Furthermore, a model’s maximum V1 neural predictivity was correlated to its V1 spatial frequency score (R = 0.565, p < 0.001). (TIFF) [file pcbi.1009739.s002.tiff]

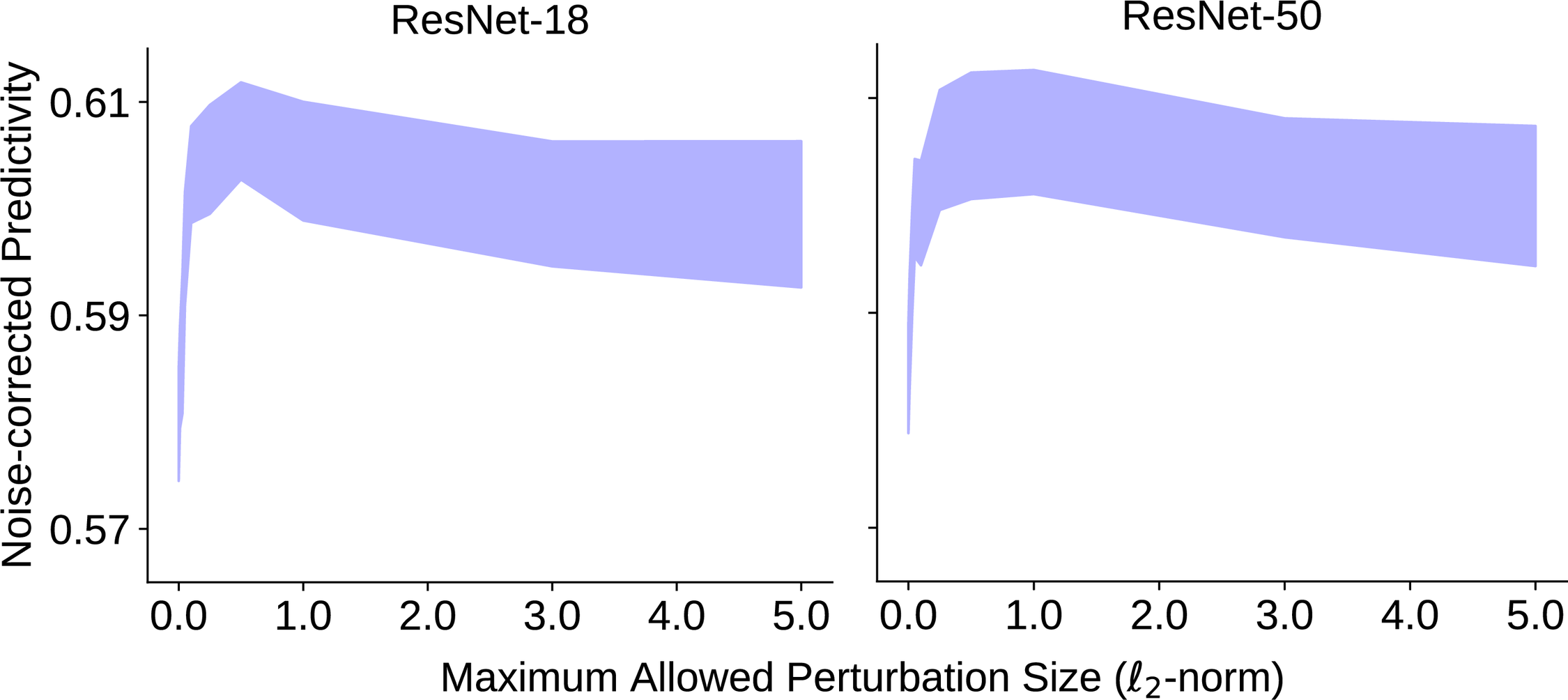

Supplement: S3 Fig — Here we asked whether V1 neural response predictivity is related to the maximum allowed size of perturbation used during adversarial training. Using previously adversarially trained models [28], we found, for both CNN architectures, that as the maximum allowed perturbation size (using the ℓ2-norm) for model training increased from zero, V1 neural predictivity increased. However, V1 neural predictivity plateaus when the ℓ2-norm of the perturbation reaches and exceeds 0.5. Thus, just increasing the maximum allowable perturbation size during adversarial training (and hence robustness to larger image perturbations) is not enough to obtain further improvements in V1 neural predictivity. (TIFF) [file pcbi.1009739.s003.tiff]

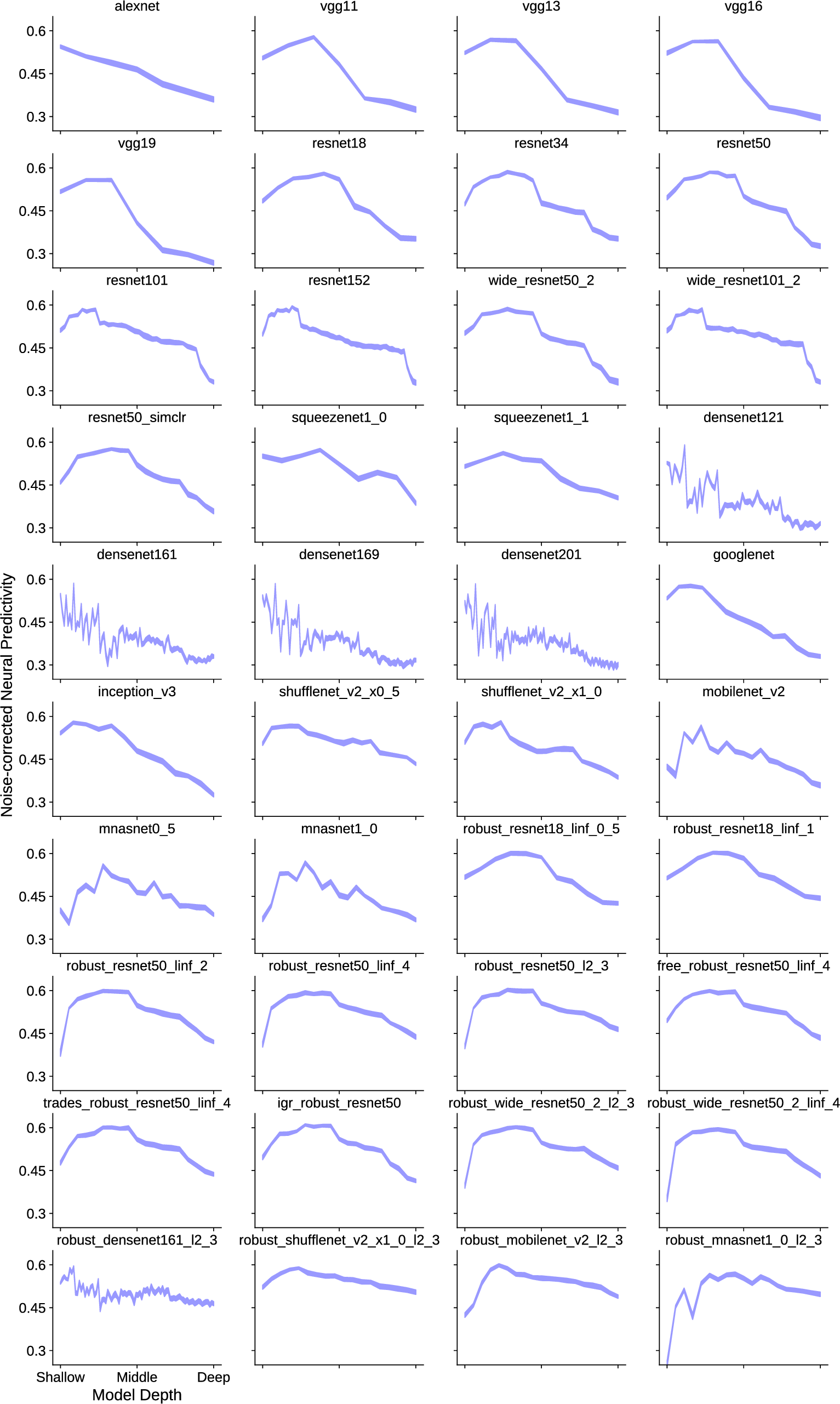

Supplement: S4 Fig — We present the neural predictivity for all 40 models as a function of its model layers. Consistent with other work [13, 14], we find that shallow to middle model layers best predict neural responses to V1 neural responses for all evaluated models. (TIFF) [file pcbi.1009739.s004.tiff]

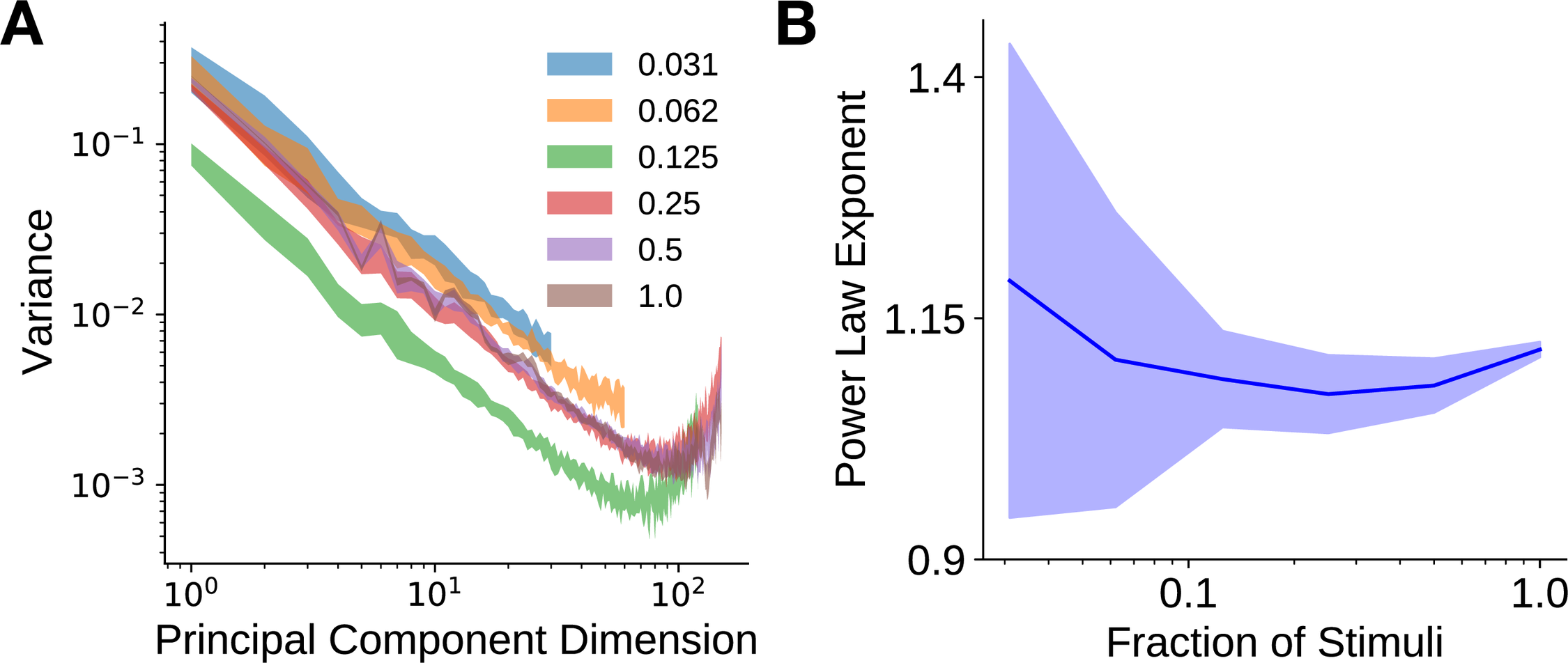

Supplement: S5 Fig — As in Stringer et al. [26], we varied the fraction of stimuli that were used in the neural response dataset and computed their eigenspectra and their associated power law exponents. For each fraction of stimuli used, we randomly sampled stimuli ten times and computed the eigenspectrum and power law exponent for each subset of the neural responses, resulting in ten power law exponents and ten eigenspectra for each fraction of stimuli. We found that for all fractions of stimuli used, the power law exponents were greater than one and were more precise as more stimuli were used. We note that the power law exponent in the macaque dataset may not have converged to one yet, so more neurons may need to be recorded in the future to further verify the power-law-like behaviour of macaque V1 neural responses. (TIFF) [file pcbi.1009739.s005.tiff]

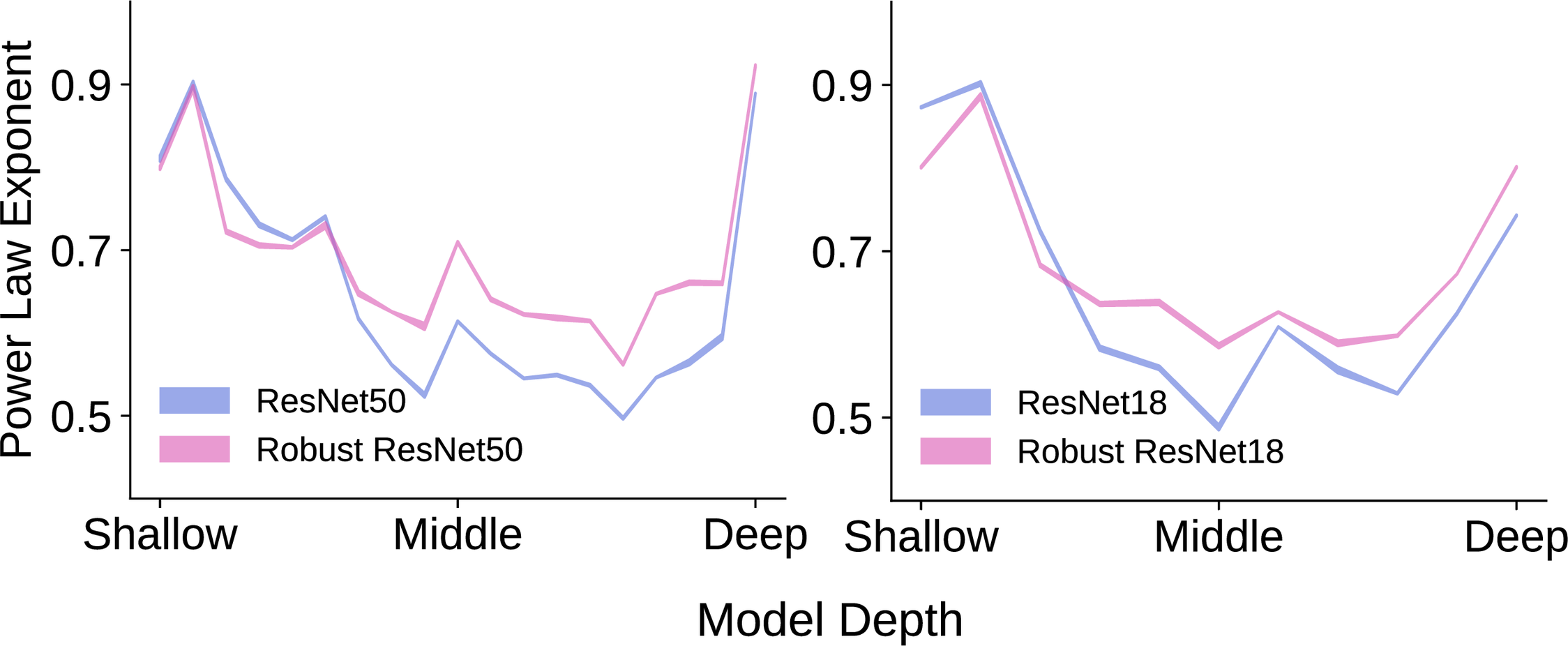

Supplement: S6 Fig — In the main text, we fit the power law exponent using principal component variances from indices 10 to 999. Here we fit the power law exponent for each eigenspectrum using principal component variances from indices 1 to 1000. This small modification to the fitting procedure does not alter the relationship between the power law exponents of robust models and those of non-robust models. Specifically, we found that the power law exponents of robust models were higher than those of non-robust models, implying that the dimensionality of the internal representations of robust models is slightly lower than that of non-robust models. (TIFF) [file pcbi.1009739.s006.tiff]

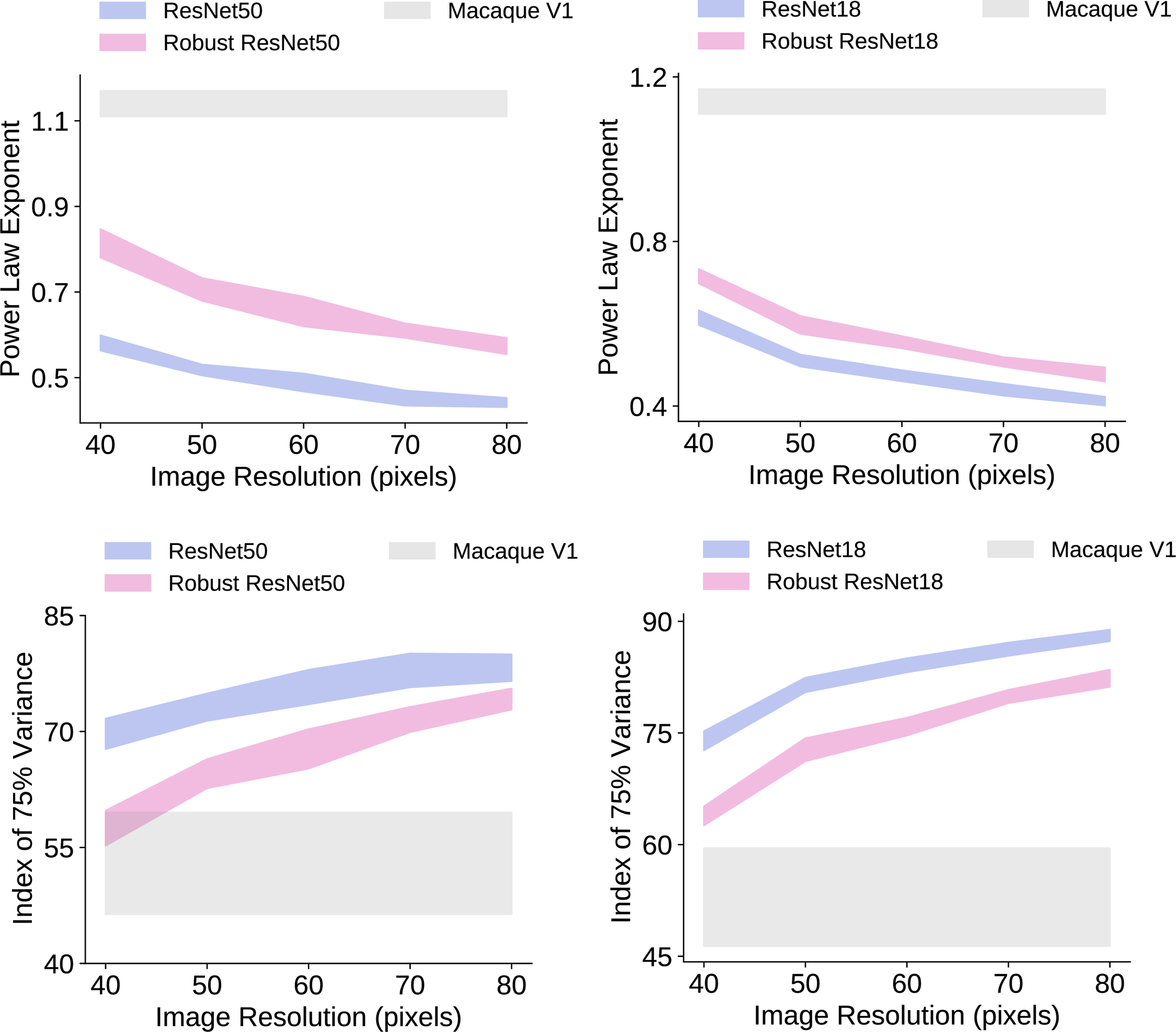

Supplement: S7 Fig — Changing the image size by downsampling the image would remove high-frequency components and thus make spectral decay steeper. Therefore, we investigated how the power law exponent varies as a function of the image resolution. We used the 1250 natural scene stimuli (which are in grayscale) from the neural response dataset of Cadena et al. [13] and varied the image resolution (in pixels) before presenting them to the models. We fixed the model architecture to be ResNet-18 and ResNet-50 (using both robust and non-robust variations of them) and varied the input resolution from 40 pixels (the size used in neural response predictions) to 80 pixels (the size of the center crop prior to the downsampling used in the neural response fitting procedure). Specifically, the image transformations were as follows: (1) Center crop the original stimulus to 80 × 80 pixels and (2) resize the image to one of {40 × 40, 50 × 50, 60 × 60, 70 × 70, 80 × 80} pixels. Using the most V1-like model layer for each of the two models, we extracted activations to the images and randomly sampled 166 artificial neurons (same as the number of neurons in the macaque V1 neural response dataset) 20 times. Using the sub-sampled model response matrix (of dimensions 1250 × 166), we computed their eigenspectra, the power law exponents and the index at which cumulative principal component variance reached 75%. This resulted in 20 power law exponents and principal component indices and the mean and the standard deviation across the 20 values was reported. We found that the power law exponents for both robust and non-robust models was lower than that of macaque V1 neural responses and that they decreased as a function of image resolution indicating that increasingly fine stimulus features are encoded as more information is available in the stimulus (top row). These observations were corroborated by another metric that measures the dimensionality of the model or the biological responses. Shown on the bottom row, [file pcbi.1009739.s007.tiff]
