# Supplementary material for: Increasing neural network robustness improves match to macaque V1 eigenspectrum, spatial frequency preference and predictivity
Source: PLoS Comput Biol. 2022 Jan 7;18(1):e1009739. doi: 10.1371/journal.pcbi.1009739 (PMC8775238; doi:10.1371/journal.pcbi.1009739)
Supplement: S1 Table — This table lists all the models we used, their macaque V1 neural response predictivity and their top-1 accuracies on ImageNet validation set images which have not been perturbed (i.e., for a perturbation, δ, and for any norm, ‖δ‖ = 0) or were adversarially perturbed with different norm constraints on the perturbations: ‖δ‖∞ ≤ 1/1020, ‖δ‖2 ≤ 0.15, ‖δ‖1 ≤ 40. For the models trained to be adversarially robust, the suffix corresponds to the norm constraint imposed on the size of the perturbation during model training. For example, robust_resnet50_l2_3 corresponds to a ResNet-50 adversarially trained to be robust to perturbations, δ, of size at most ‖δ‖2 ≤ 3 [28], igr_robust_resnet50 corresponds to a ResNet-50 trained with input gradient regularization (IGR, [21]) and resnet50_simclr corresponds to a ResNet-50 trained with the SimCLR unsupervised loss function [47]. (PDF) [file pcbi.1009739.s008.pdf]

| **Model Identifier** | $\|\boldsymbol{\delta}\| = 0$ | $\|\boldsymbol{\delta}\|_\infty \leq 1/1020$ | $\|\boldsymbol{\delta}\|_2 \leq 0.15$ | $\|\boldsymbol{\delta}\|_1 \leq 40$ | **Neural Predictivity** |
|---|---|---|---|---|---|
| `alexnet` | 0.565 | 0.294 | 0.391 | 0.383 | 0.545 |
| `vgg11` | 0.690 | 0.264 | 0.401 | 0.376 | 0.578 |
| `vgg13` | 0.699 | 0.183 | 0.308 | 0.271 | 0.569 |
| `vgg16` | 0.716 | 0.211 | 0.330 | 0.281 | 0.563 |
| `vgg19` | 0.724 | 0.228 | 0.340 | 0.287 | 0.558 |
| `resnet18` | 0.698 | 0.188 | 0.344 | 0.327 | 0.580 |
| `resnet34` | 0.733 | 0.227 | 0.385 | 0.364 | 0.586 |
| `resnet50` | 0.761 | 0.230 | 0.379 | 0.352 | 0.585 |
| `resnet101` | 0.774 | 0.253 | 0.404 | 0.375 | 0.584 |
| `resnet152` | 0.783 | 0.284 | 0.429 | 0.394 | 0.594 |
| `wide_resnet50_2` | 0.785 | 0.297 | 0.439 | 0.409 | 0.586 |
| `wide_resnet101_2` | 0.788 | 0.297 | 0.436 | 0.403 | 0.584 |
| `squeezenet1_0` | 0.581 | 0.166 | 0.279 | 0.254 | 0.573 |
| `squeezenet1_1` | 0.582 | 0.128 | 0.220 | 0.177 | 0.562 |
| `densenet121` | 0.744 | 0.191 | 0.348 | 0.331 | 0.586 |
| `densenet161` | 0.771 | 0.244 | 0.399 | 0.375 | 0.582 |
| `densenet169` | 0.756 | 0.204 | 0.361 | 0.342 | 0.581 |
| `densenet201` | 0.769 | 0.281 | 0.436 | 0.415 | 0.580 |
| `googlenet` | 0.698 | 0.260 | 0.399 | 0.381 | 0.577 |
| `inception_v3` | 0.772 | 0.333 | 0.514 | 0.549 | 0.578 |
| `mobilenet_v2` | 0.719 | 0.114 | 0.251 | 0.213 | 0.562 |
| `mnasnet0_5` | 0.677 | 0.174 | 0.292 | 0.259 | 0.557 |
| `mnasnet1_0` | 0.735 | 0.129 | 0.269 | 0.227 | 0.567 |
| `shufflenet_v2_x0_5` | 0.606 | 0.110 | 0.185 | 0.133 | 0.566 |
| `shufflenet_v2_x1_0` | 0.694 | 0.093 | 0.182 | 0.140 | 0.579 |
| `robust_resnet50_l2_3` | 0.628 | 0.612 | 0.618 | 0.617 | 0.603 |
| `robust_resnet50_linf_4` | 0.639 | 0.626 | 0.611 | 0.518 | 0.593 |
| `robust_resnet50_linf_2` | 0.691 | 0.671 | 0.661 | 0.598 | 0.599 |
| `robust_resnet18_linf_1` | 0.635 | 0.602 | 0.610 | 0.600 | 0.603 |
| `robust_resnet18_linf_0_5` | 0.661 | 0.614 | 0.627 | 0.614 | 0.601 |
| `robust_wide_resnet50_linf_4` | 0.684 | 0.670 | 0.659 | 0.594 | 0.594 |
| `robust_wide_resnet50_l2_3` | 0.669 | 0.652 | 0.658 | 0.657 | 0.602 |
| `robust_densenet161_l2_3` | 0.670 | 0.651 | 0.658 | 0.656 | 0.591 |
| `robust_mnasnet1_0_l2_3` | 0.410 | 0.388 | 0.398 | 0.398 | 0.567 |
| `robust_mobilenet_v2_l2_3` | 0.496 | 0.479 | 0.486 | 0.485 | 0.598 |
| `robust_shufflenet_v2_x1_0_l2_3` | 0.433 | 0.417 | 0.422 | 0.421 | 0.588 |
| `free_robust_resnet50_linf_4` | 0.604 | 0.587 | 0.576 | 0.512 | 0.599 |
| `trades_robust_resnet50_linf_4` | 0.655 | 0.559 | 0.570 | 0.544 | 0.601 |
| `igr_robust_resnet50` | 0.671 | 0.623 | 0.641 | 0.637 | 0.610 |
| `resnet50_simclr` | 0.678 | 0.079 | 0.185 | 0.171 | 0.576 |
