# Supplementary material for: Increasing neural network robustness improves match to macaque V1 eigenspectrum, spatial frequency preference and predictivity
Source: PLoS Comput Biol. 2022 Jan 7;18(1):e1009739. doi: 10.1371/journal.pcbi.1009739 (PMC8775238; doi:10.1371/journal.pcbi.1009739)
Supplement: S1 Appendix — Here we provide additional details on adversarial training and on generating adversarial examples. (PDF) [file pcbi.1009739.s009.pdf]

The steps to generate adversarial examples from the original image, $\boldsymbol{x}$, are as follows:

$$\begin{aligned}
\boldsymbol{\delta}^{(t)} &= \mathsf{Step}\left(\nabla_{\boldsymbol{v}^{(t)}}\mathcal{L}(\boldsymbol{v}^{(t)};\boldsymbol{\theta})\right) \\
\boldsymbol{v}^{(t+1)} &= \boldsymbol{v}^{(t)} + \eta\boldsymbol{\delta}^{(t)} \\
\boldsymbol{v}^{(t+1)} &= \boldsymbol{x} + \mathsf{Project}\left(\boldsymbol{v}^{(t+1)} - \boldsymbol{x}, \varepsilon\right) && \text{(ensure perturbation satisfies constraint)} \\
\boldsymbol{v}^{(t+1)} &= \max\left(\min\left(\boldsymbol{v}^{(t+1)}, 1\right), 0\right), && \text{(clip perturbed image)}
\end{aligned}$$

where $\mathcal{L}(\cdot;\boldsymbol{\theta})$ is the cross-entropy loss, η is the gradient ascent step size, $\boldsymbol{v}^{(t)}$ is the perturbed image at step t of projected gradient ascent and is initialized to the original image (i.e., $\boldsymbol{v}^{(0)} = \boldsymbol{x}$) and $\boldsymbol{\theta}$ are the model parameters. Depending on the norm constraint on the perturbations, $\mathsf{Step}(\cdot)$ and $\mathsf{Project}(\cdot,\cdot)$ would be implemented differently.

For ℓ_∞-norm constraints, $\mathsf{Step}(\cdot)$ computes the sign of the gradient and $\mathsf{Project}(\cdot,\cdot)$ clamps the perturbation to be within $[-\varepsilon, \varepsilon]$:

$$\begin{aligned}
\mathsf{Step}\left(\boldsymbol{g}\right) &= \operatorname{sign}(\boldsymbol{g}), \\
\mathsf{Project}\left(\boldsymbol{\delta}, \varepsilon\right) &= \max\left(\min\left(\boldsymbol{\delta}, \varepsilon\right), -\varepsilon\right).
\end{aligned}$$

For ℓ_2-norm constraints, $\mathsf{Step}(\cdot)$ normalizes the gradient (so that $\|\boldsymbol{g}\|_2 = 1$) and $\mathsf{Project}(\cdot,\cdot)$ ensures that the ℓ_2-norm of the perturbation does not exceed ε:

$$\begin{aligned}
\mathsf{Step}\left(\boldsymbol{g}\right) &= \boldsymbol{g}/\|\boldsymbol{g}\|_2, \\
\mathsf{Project}\left(\boldsymbol{\delta}, \varepsilon\right) &= \begin{cases} \varepsilon \cdot \boldsymbol{\delta}/\|\boldsymbol{\delta}\|_2, & \text{if } \|\boldsymbol{\delta}\|_2 > \varepsilon \\ \boldsymbol{\delta}, & \text{otherwise} \end{cases}
\end{aligned}$$

$\mathsf{Step}(\cdot)$ and $\mathsf{Project}(\cdot,\cdot)$ for ℓ_1-norm constraints are similarly defined to those of ℓ_2-norm constraints (by replacing $\|\cdot\|_2$ with $\|\cdot\|_1$).
